# Predicting crown width using nonlinear mixed-effects models accounting for competition in multi-species secondary forests

**DOI:** 10.7717/peerj.13105

**Published:** 2022-04-27

**Authors:** Rui Hou, Zongzheng Chai

**Affiliations:** College of Forestry, Guizhou University, Guiyang, Guizhou, China

**Keywords:** Spatially non-explicit models, Spatially explicit models, Hegyi index, Forest growth model

## Abstract

Crown width (CW) is an important tree variable and is often used as a covariate predictor in forest growth models. The precise measurement and prediction of CW is therefore critical for forest management. In this study, we introduced tree species as a random effect to develop nonlinear mixed-effects CW models for individual trees in multi-species secondary forests, accounting for the effects of competition. We identified a simple power function for the basic CW model. In addition to diameter at breast height (DBH), other significant predictor variables including height to crown base (HCB), tree height (TH), and competition indices (CI) were selected for the mixed-effects CW model. The sum of relative DBH (SRD) was identified the optimal distance-independent CI and as a covariate predictor for spatially non-explicit CW models, whereas the sum of the Hegyi index for fixed number competitors (SHGN) was the optimal distance-dependent CI for spatially explicit CW models, with significant linear correlation (*R*^2^ = 0.943, *P* < 0.001). Both spatially non-explicit and spatially explicit mixed-effects CW models were developed for studied secondary forests. We found that these models can describe more than 50% of the variation in CW without significant residual trends. Spatially explicit models exhibited a significantly larger effect on CW than spatially non-explicit ones; however, spatially explicit models are computationally complex and difficult and can be replaced by corresponding spatially non-explicit models due to the small differences in the fit statistics. The models we present may be useful for forestry inventory practices and have the potential to aid the evaluation and management of secondary forests in the region.

## Introduction

Crown width (CW) is the horizontal distance passing through the center of the tree trunk from the points of crown azimuth (Sharma et al., 2017). Variation in CW is largely the result of spacing, and is frequently used to estimate the vigor, growth and competition of trees. The identification and estimation of CW can also be used to calculate stand canopy closure, which is important for assessing wildlife habitat suitability, fire risk, and understory light conditions for regeneration (Fu et al., 2017; Yang & Huang, 2017). Consequently, the quantification of CW is an important component of many forest growth and yield models (Russell & Weiskittel, 2011; Wang et al., 2017; Wang et al., 2020). The measurement of CW is uncommon in forest inventories, yet its value is widely applicable in forestry (Russell & Weiskittel, 2011; Raptis et al., 2018). In tall, closed-canopy and dense stands, especially multi-species mixed forests, CW is less accurate and often far more difficult and time consuming to measure. Therefore, allometric models of CW based on a few trees are commonly used to predict the CW of remaining trees, thus reducing the cost of data acquisition (Fu et al., 2013; Buchacher & Ledermann, 2020).

Competition is an important ecological factor affecting the growth of individual trees or stands (Dolezal et al., 2009; Fichtner et al., 2012), with tree crown morphology reflecting the cumulative competition effects over time that are central to many aspects of forest ecology (Sharma, Vacek & Vacek, 2016; Yang & Huang, 2017). There is ample evidence of tree crown plastic responses to inter-tree competition, which is reduced by crown plasticity (Longuetaud et al., 2013). Thus, tree crown growth is significantly affected by competition, which directly or indirectly affects tree survival and population dynamics, as well as the CW of individual trees (Aakala, Berninger & Starr, 2018; Wang et al., 2020). Models of CW are often a function of commonly measured stand and tree attributes, however, evidence has shown that crown widths are often over-predicted in dense stands and under-predicted in sparse stands when CW is modelled using tree size alone, and competition effects need to be considered for more accurate CW models (Buchacher & Ledermann, 2020).

The karst region of Southwest China is one of the three largest concentrated karst areas in the world (Wang et al., 2019). This region, which has suffered severe environmental degradation, features diverse karst types. Rock desertification has been severe, and vegetation restoration has been widely implemented to reverse the environmental degradation caused by human activities (Chai et al., 2018; Chi et al., 2020). Secondary forests are a typical vegetation type in the area, and exhibit a broad ecological amplitude, as they have formed with varying patterns of natural succession after a long period of natural recovery. Modelling tree CW for these secondary forests could greatly increase our understanding of stand dynamics and the capacity of these forests for ecological restoration, as well as help achieve a variety of management objectives. However, few CW models for secondary forests have been developed for karst regions, and all of these models use diameter as a single predictor, disregarding competition among trees and therefore having relatively low accuracy and limited scope of application (Yuan et al., 2017).

We hypothesized that the CW model for secondary forests would be significantly influenced by competition. To evaluate this hypothesis, we developed mixed-effects CW models for secondary forests, incorporating a competition factor. The objectives of this study were: (1) to examine the effects of distance-dependent and distance-independent CIs on the CW model; and (2) to develop mixed-effects models for precise CW predictions of multi-species secondary forests in study area.

## Materials and Methods

### Study site

Guizhou Province is located in the center of the Southeast Asian Karst Region, which is the largest karst area in the world. Karstification is highly developed in this area and the karst types are the most diverse of any karst area globally (Chai et al., 2018). The study took place in the Ziyun Miao and Buyi Autonomous County (105°55′14″–106°29′56″E, 25°21′43″–26°2′30″N) in southwestern Guizhou Province. The region is a typical karst region that has high biodiversity and abundant forest resources, and experiences a mild and humid continental monsoon climate with a mean altitude of 1,185 m, mean annual temperature of 15.30 °C and mean annual rainfall of 1,337.10 mm.

Our study site was the Zhongdong Scenic Area of the Getuanhe National Scenic and Historic Interest Area in Ziyun Miao and Buyi autonomous County, which is a typical karst landform with non-zonal limestone soil and a shallow soil layer. The parent material of the soil is mainly sedimentary rock and carbonate rock. Prior to abandonment in the mid-1980s, cultivated land in this region was extensive and widely distributed. Corn was once the main crop, but after 30 years of reduced human disturbance and reasonable natural recovery, much of the area is now covered by secondary forest and plantation (Chi et al., 2020).

### Data collection

**Figure 1:**
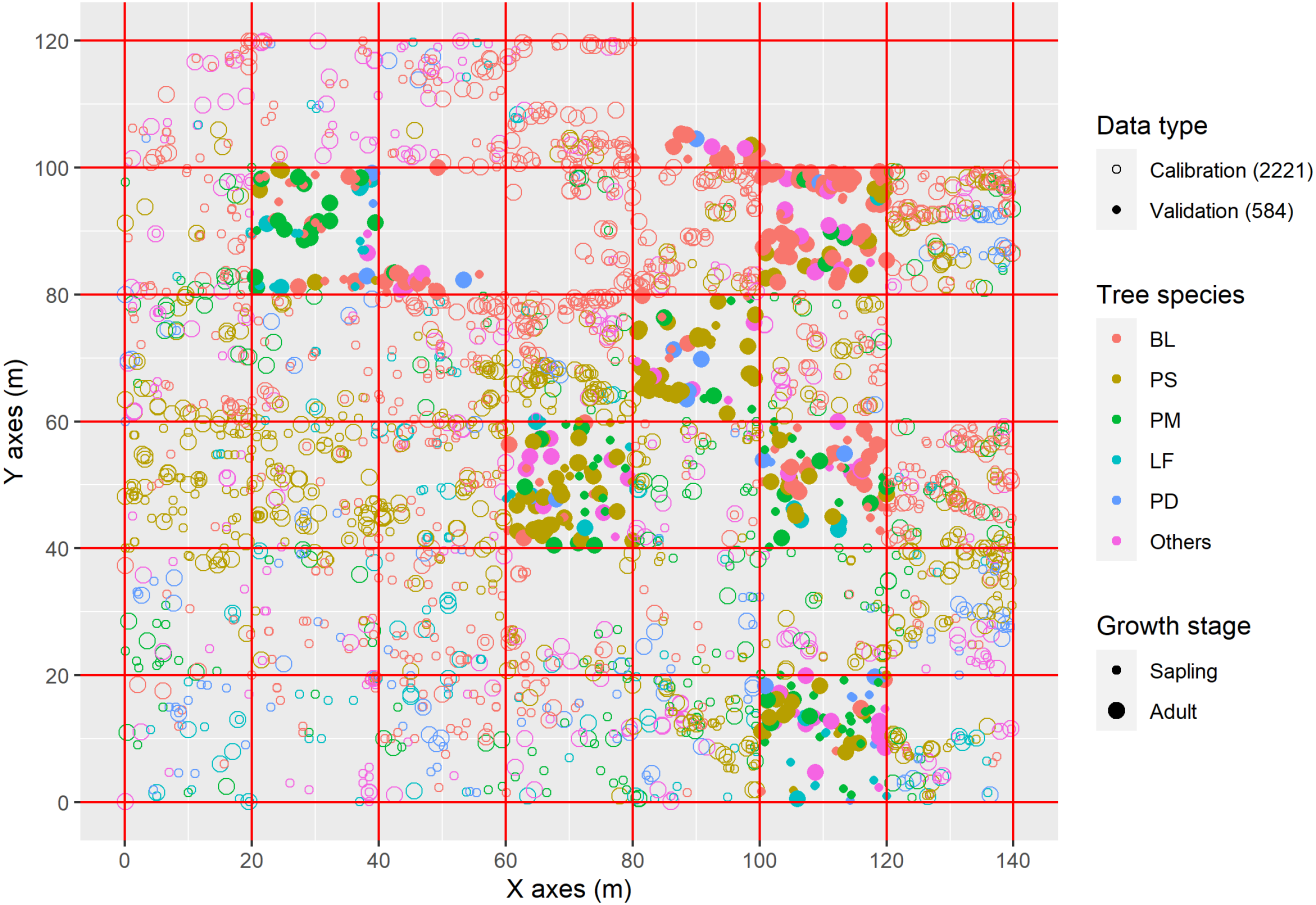
Distribution of tree species in sampling plot. The trees were roughly divided into two growth stages, saplings (1 cm ≤ DBH < 5 cm) and adults (DBH ≥ 5 cm); BL, *Betula luminifera*; PS, *Platycarya strobilacea*; PM, *Pinus massoniana*; LF, *Liquidambar formosana*; PD, *Populus davidiana ; Others:* other species except BL, PS, PM, LF, and PD.

Following field reconnaissance according to the field protocol of the Center for Tropical Forest Science (CTFS) (Condit, 1998), we established an area of 0.168 km^2^ (120 × 140 m) as a permanent sampling plot with typical and low disturbance. The large sample plot was further divided into 42 plots (20 × 20 m) (Fig. 1). In each plot, the altitude, slope gradient and slope aspect were recorded, all woody plants were surveyed and identified, and trees with a diameter at breast height (DBH, at 1.3 m) ≥1 cm were marked. The parameters of species, tree height, DBH, height of crown base, crown width, and crown condition of the trees were determined, and crown width was taken as the arithmetic mean of two crown widths as obtained from measurements of four crown radii in four directions representing two perpendicular azimuths. The geographic coordinates of the trees were recorded with the southwestern corner of the plot taken as the origin (Chi et al., 2020). The trees were roughly divided into two growth stages, saplings (1 cm ≤ DBH < 5 cm) and adults (DBH ≥ 5 cm), and tree species were classified into six groups according to the most numerous species: *Betula luminifera* (BL), *Platycarya strobilace* a (PS), *Pinus massoniana* (PM), *Liquidambar formosana* (LF), *Populus davidiana* (PD) and others. Only 39 of 42 plots contained trees, and those 39 plots were randomly divided in two groups: 31 subplots (80%) contained 2,221 trees for calibration data, while validation was performed using 584 trees in the remaining eight plots. The distribution of trees and plots is shown in Fig. 1.

### Data analysis

#### CW-D candidate function

Twelve candidate nonlinear CW-D functions (see Table 1) were selected from previous studies based on the suitability of their mathematical features, the possible biological interpretation of model parameters, and satisfactory predictions of the CW-D relationship in the literature (Fu et al., 2013; Raptis et al., 2018; Wang et al., 2021), and the best fitting performance model was named as the basic CW model for studied secondary forest.

**Table 1:** Crown width-DBH (CW-D) candidate functions considered in the analysis (Fu et al., 2013; Raptis et al., 2018; Wang et al., 2021).

ID	Function	Function form
F1	CW = *ϕ*1 + *ϕ*2D	Linear
F2	CW = *ϕ*1D^*ϕ*2^	Power
F3	CW = *ϕ*1[1 − exp(−*ϕ*2D)]	Monomolecular
F4	CW = [D/(*ϕ*1 + *ϕ*2D)]^2^	Hossfeld 1
F5	CW = *ϕ*1(*ϕ*2)^D^	Compound
F6	CW = exp(*ϕ*1 + *ϕ*2D)	Growth
F7	CW = *ϕ*1exp(*ϕ*2D)	Exponential
F8	CW = *ϕ*1 + *ϕ*2D + *ϕ*3D^2^	Quadratic
F9	CW = *ϕ*1[1 − exp(−*ϕ*2D)]^*ϕ*3^	Richards
F10	CW = *ϕ*1/[1 + *ϕ*2exp(−*ϕ*3D)]	Logistic
F11	$\mathrm{CW}=\phi 1/[1+\exp (\phi 2+\phi 3\ln \left( \mathrm{D}+1 \right) )]$	Logistic
F12	CW = *ϕ*1[1 − exp(−*ϕ*2D^*ϕ*3^)]	Weibull

#### Selection of variables

In addition to DBH, a number of tree and stand variables have been reported to correlate highly with CW, namely tree size, stand level, and competition (Sharma, Vacek & Vacek, 2016). The candidate variables selected during the development stage of a generalized nonlinear crown prediction model were (1) tree size variables, such as tree height (TH), height of crown base (HCB); (2) stand level variables, such as stand density (DEN), mean TH of stand (MTH), mean dominant TH of stand (MDH), species Shannon diversity of stand (SHN); and (3) competition measures; six competition indices including four distance-independent indices and two distance-dependent indices were compared for their performance in predicting crown width (Table 2). For distance-dependent indices, identifying competitor trees are of great importance to derive competition indices. Generally, studies either identify four neighboring trees (four neighboring trees as competitors in four different directions around the subject tree) or use fixed radii (competitors are identified as all neighboring trees around the subject tree within a search radius of 3.5 times the mean crown radius of canopy trees) (Wang et al., 2021); in our study, the relevant radius is 5 m. We applied these two methods to calculate the Hegyi indices SHGR and SHGN, respectively (Table 2). The crown width for stand-growth trees is significantly influenced by competition among individual trees, the Hegyi index is based on the principle that larger and closer competitor would have higher competition impact on a subject tree (Sharma, Vacek & Vacek, 2016), has been being developed since the 1970s to quantify individual-tree-level competition (Hegyi, 1974) and frequently used to develop various spatially explicit forest models including crown models (Pommerening, 2008; Sharma, Vacek & Vacek, 2016). To eliminate edge effects and improve the accuracy of the distance-dependent indices, we made 8 copies of the sample plot, and then moved one of each copy from its original position into up, down, left, right, upper left, lower left, lower right, and upper right directions, respectively. Thus a new, larger sample plot consisting of 9 copies is created; this edge correction can individually evaluate each tree to determine whether all nearest neighbors are truly located within the plot (Wang et al., 2021). For summary statistics of studied variables, see Tables 3 and 4.

**Table 2:** Competition index selected for this study.

Item	Indices	Abb.	Formula
Distance-independent	Sum of the DBH of all trees per plot	SD	${\mathop{\sum }\nolimits }_{i=1}^{n}{D}_{i}$
	Sum of the DBH of dominant trees per plot	SDD	${\mathop{\sum }\nolimits }_{i=1}^{n}D{D}_{i}$
	Sum of the relative DBH of all tree per plot	SRD	${\mathop{\sum }\nolimits }_{i=1}^{n} \frac{{D}_{i}}{MDD} $
	Sum of the basal area of all trees per plot	SBA	${\mathop{\sum }\nolimits }_{i=1}^{n}B{A}_{i}$
Distance-dependent	Sum of Hegyi indices for fixed number competitors of all trees per plot	SHGN	${\mathop{\sum }\nolimits }_{i=1}^{n}{\mathop{\sum }\nolimits }_{j=1}^{m} \frac{{D}_{j}}{{D}_{i}\cdot ({L}_{ij}+1)} $
	Sum of Hegyi indices for fixed radii of all trees per plot	SHGR	

**Notes.**

MDD is mean dominant DBH of stand.

**Table 3:** Summary statistics for tree variables of different tree species.

Tree species	Abbr.	Calibration				Validation			
		Min.	Max.	Mean	SD	Min.	Max.	Mean	SD
*Betula luminifera* (BL)	CW	0.36	8.70	2.90	1.49	0.85	8.05	2.88	1.51
	DBH	2.30	46.30	7.22	4.10	2.30	19.20	6.19	3.24
	TH	2.50	16.90	8.54	2.72	2.00	16.70	8.01	2.46
	HCB	0.20	13.80	4.62	2.18	0.20	15.00	4.40	1.99
*Platycarya strobilacea* (PS)	CW	0.44	8.30	2.32	0.96	0.60	7.45	2.57	1.08
	DBH	2.00	29.40	5.12	2.25	2.60	15.10	4.89	1.78
	TH	2.60	14.80	6.66	2.09	2.50	15.60	6.90	1.84
	HCB	0.40	9.50	3.11	1.65	0.50	6.30	2.69	1.16
*Pinus massoniana* (PM)	CW	0.84	4.85	2.48	0.74	0.80	6.60	2.87	1.11
	DBH	2.30	20.90	6.49	3.22	2.80	16.70	7.26	4.02
	TH	1.50	11.70	5.84	1.91	2.60	13.40	7.09	2.48
	HCB	0.20	6.40	1.79	1.30	0.20	4.90	1.59	0.83
*Liquidambar formosana* (LF)	CW	0.36	9.65	3.48	1.63	1.30	8.68	3.68	1.57
	DBH	1.20	26.30	8.97	5.54	2.90	36.10	8.13	5.70
	TH	2.80	18.20	8.89	3.53	3.00	21.30	9.02	3.34
	HCB	0.30	15.00	2.71	2.13	0.40	7.70	2.69	1.63
*Populus davidiana* (PD)	CW	0.68	7.75	2.62	0.98	0.55	5.41	2.63	1.37
	DBH	2.90	21.10	6.25	3.07	3.00	23.10	5.89	3.82
	TH	3.00	14.50	7.66	2.55	3.80	17.30	7.79	2.60
	HCB	0.50	8.90	3.22	1.52	1.40	6.30	3.51	1.27
Others	CW	0.24	6.55	2.28	0.95	0.52	8.15	2.06	1.17
	DBH	1.00	21.80	5.45	2.70	2.40	14.10	4.57	2.34
	TH	1.70	18.30	5.67	2.16	2.30	11.30	5.72	1.82
	HCB	0.20	9.20	2.49	1.59	0.40	8.60	2.48	1.55

**Table 4:** Summary statistics for tree, stand and competition variables of studied forest.

Item	Abbr.	Calibration				Validation			
		Min.	Max.	Mean	SD	Min.	Max.	Mean	SD
Tree	CW	0.24	9.65	2.64	1.26	0.52	8.68	2.75	1.35
	DBH	1.00	46.30	6.39	3.65	2.30	36.10	5.95	3.41
	TH	1.50	18.30	7.36	2.73	2.00	21.30	7.37	2.47
	HCB	0.20	15.00	3.46	2.10	0.20	15.00	3.11	1.84
Stand	DEN	825	3550	1877	813	600	2625	1825	791
	MTH	6.22	9.44	7.44	0.82	5.86	8.43	7.46	0.82
	MDH	9.24	15.60	12.17	1.68	7.88	18.07	11.50	3.11
	SHN	0.78	2.16	1.48	0.37	0.79	2.01	1.33	0.40
Competition	SD	239.30	805.50	457.69	159.94	123.40	645.90	434.09	206.30
	SDD	303.03	1525.18	804.64	360.85	177.60	2055.90	813.31	613.27
	SRD	19.56	100.12	42.02	17.77	16.68	76.31	44.45	22.34
	SBA	0.11	0.64	0.30	0.13	0.06	0.54	0.27	0.18
	SHGR	12.61	47.99	25.29	10.51	7.79	35.22	25.65	11.12
	SHGN	15.67	86.22	40.30	21.17	12.47	57.17	41.87	18.21

### Mixed effect models

The mixed-effects model is a model that includes both fixed- and random-effect variables. In this study, a nonlinear mixed-effects (NLME) modeling framework was used for the hierarchical structure of CW-D data, we set tree species as a random effect. Numerous studies have applied mixed-effects models to describe CW–D relationships and have improved model fit and prediction accuracy. At the plot level, the NLME model of the *j* th tree height in the *i* th sample plot was modeled as:

(1) \begin{eqnarray*} \left\{ \begin{array}{@{}l@{}} \displaystyle {\mathrm{CW}}_{ij}=f \left( {\phi }_{ij},{u}_{ij} \right) +{}_{ij}; \\ \displaystyle i=1,2,3,\ldots ,m;j=1,2,3,\ldots ,{n}_{i} \end{array} \right. \end{eqnarray*}

where CW_*ij*_represents the CW of the *j* th tree in the *i* th plot; *m* and *n*_*i*_ are the number of plots and observations in the *i* th plot, respectively; *f* (.) is a real-valued and differentiable function of a group-specific parameter vector *ϕ*_*ij*_ and covariate vector *u*_*ij*_; and *ɛ*_*ij*_ is a within-group error, which subjects to a multivariate normal distribution with a mean value vector of 0 and variance–covariance matrix of *R*. *ϕ*_*ij*_ is given as:

(2) \begin{eqnarray*}{\phi }_{ij}={A}_{ij}\beta +{B}_{ij}{b}_{i};{b}_{i}\sim N(0,G)\end{eqnarray*}

where *β* is the fixed-effect parameter vector; *b*_*i*_ is the random-effect parameter vector of the *i* th plot, which was assumed to have a multivariate normal distribution with a mean value vector of 0 and variance–covariance matrix of *G*, *A*_*ij*_, and *B*_*ij*_ are the incidence matrices of the appropriate dimensions, consisting of 0 or 1. Variance heterogeneity was removed by three frequently used variance functions: the exponential function, power function, and the constant plus power function, and the most effective variance functions was determined by AIC (Fu et al., 2013).

### Model evaluation

The following five statistical indices describing model performance were used in this study to evaluate the fit of the CW-D models: Akaike information criterion (AIC), coefficient of determination (R_*a*_^2^), root mean square error (RMSE), mean absolute prediction error (MAE), and mean absolute percentage error (MAPE). The expressions of these statistical criteria are summarized in Table 5. In general, models with the lowest AIC, RMSE, RMA, MAPE and with the highest R_*a*_^2^ are known to have the best performance.

**Table 5:** Model performance criteria selected for this study.

ID	Function name	Equation
1	Akaike’s information criterion (AIC)	$AIC=-2\ln \left( L \right) +2p$
2	Adjusted determinant coefficient (R_*a*_^2^)	${R}_{a}^{2}=1- \frac{n-1}{n-p-1} \frac{{\mathop{\sum }\nolimits }_{i=1}^{n}({H}_{i}-{\hat {H}}_{i})^{2}}{{\mathop{\sum }\nolimits }_{i=1}^{n}({H}_{i}-\overline{H})^{2}} $
3	root mean square error (RMSE)	$RMSE=\sqrt{ \frac{{\mathop{\sum }\nolimits }_{i=1}^{n}({H}_{i}-{\hat {H}}_{i})^{2}}{n} }$
4	mean absolute prediction error (MAE)	$MAE= \frac{{\mathop{\sum }\nolimits }_{i=1}^{n} \left\vert {H}_{i}-{\hat {H}}_{i} \right\vert }{n} $
5	Mean absolute percentage error (MAPE)	$MAPE= \frac{{\mathop{\sum }\nolimits }_{i=1}^{n} \frac{ \left\vert {H}_{i}-{\hat {H}}_{i} \right\vert }{{H}_{i}} }{n} $

**Notes.**

Note: *H*_*i*_ is the observed value, ${\hat {H}}_{i}$ is the predicted value, $\overline{H}$ is the mean observed value, *n* is the number of observations used for fitting the model, and ln is the natural logarithm, *L* is the likelihood function, *p* is the number of model parameters to be estimated.

**Table 6:** Crown width-DBH (CW–D) functions and goodness-of-fit statistics in the analysis.

ID	Calibration					Validation			
	AIC	${R}_{a}^{2}$	RMSE	MAE	MAPE	${R}_{a}^{2}$	RMSE	MAE	MAPE
F1	5998	0.453	0.932	0.684	0.869	0.399	1.066	0.789	1.136
F2	**5969**	**0.460**	**0.926**	**0.686**	**0.858**	**0.416**	**1.054**	**0.781**	**1.110**
F3	6019	0.448	0.937	0.698	0.878	0.419	1.060	0.784	1.124
F4	6071	0.435	0.948	0.705	0.899	0.416	1.061	0.786	1.126
F5	6388	0.348	1.018	0.733	1.036	0.309	1.128	0.849	1.273
F6	6388	0.348	1.018	0.733	1.036	0.309	1.128	0.849	1.273
F7	6388	0.348	1.018	0.733	1.036	0.309	1.128	0.849	1.273
F8	5950	0.465	0.922	0.684	0.850	0.412	1.056	0.782	1.115
F9	5970	0.460	0.926	0.686	0.858	0.416	1.053	0.781	1.109
F10	5951	0.465	0.922	0.682	0.850	0.402	1.063	0.786	1.130
F11	−	−	−	−	−	−	−	−	−
F12	5971	0.460	0.926	0.686	0.858	0.416	1.053	0.781	1.109

### Statistical analysis

All statistical analyses were performed using R version 4.0.3. Regression was executed using the minpack.lm and nlme package. The figures were drawn and the data were manipulated using the ggplot2 and plyr packages, respectively.

## Results

### Basic CW model

In the comparison of goodness-of-fit statistics for the 12 CW-D functions fitted to the data for the secondary forests (Table 6), all showed fluctuations within a relatively small range except the Logistic (F11) function, which did not converge, and their parameter estimates were significant at the *P* < 0.001 level. The Power (F2), Logistic (F10) and Quadratic (F8) functions were found to be superior to other candidate functions during the calibration stage, and the F2 functions performed better than the F8 and F10 during the validation stage. Therefore, the Power (F2) function was selected as the basic CW model in this study. It is expressed as follows:

(3) \begin{eqnarray*}{\mathrm{CW}}_{ij}=0.861{\mathrm{D}}_{ij}^{0.620}+{}_{ij}\end{eqnarray*}

where CW_*ij*_ and D_*ij*_ represent the crown width (m) and DBH (cm) of the *j* th tree in the *i* th plot, respectively, and *ɛ*_*ij*_ is the error term.

### Generalized CW model

**Figure 2:**
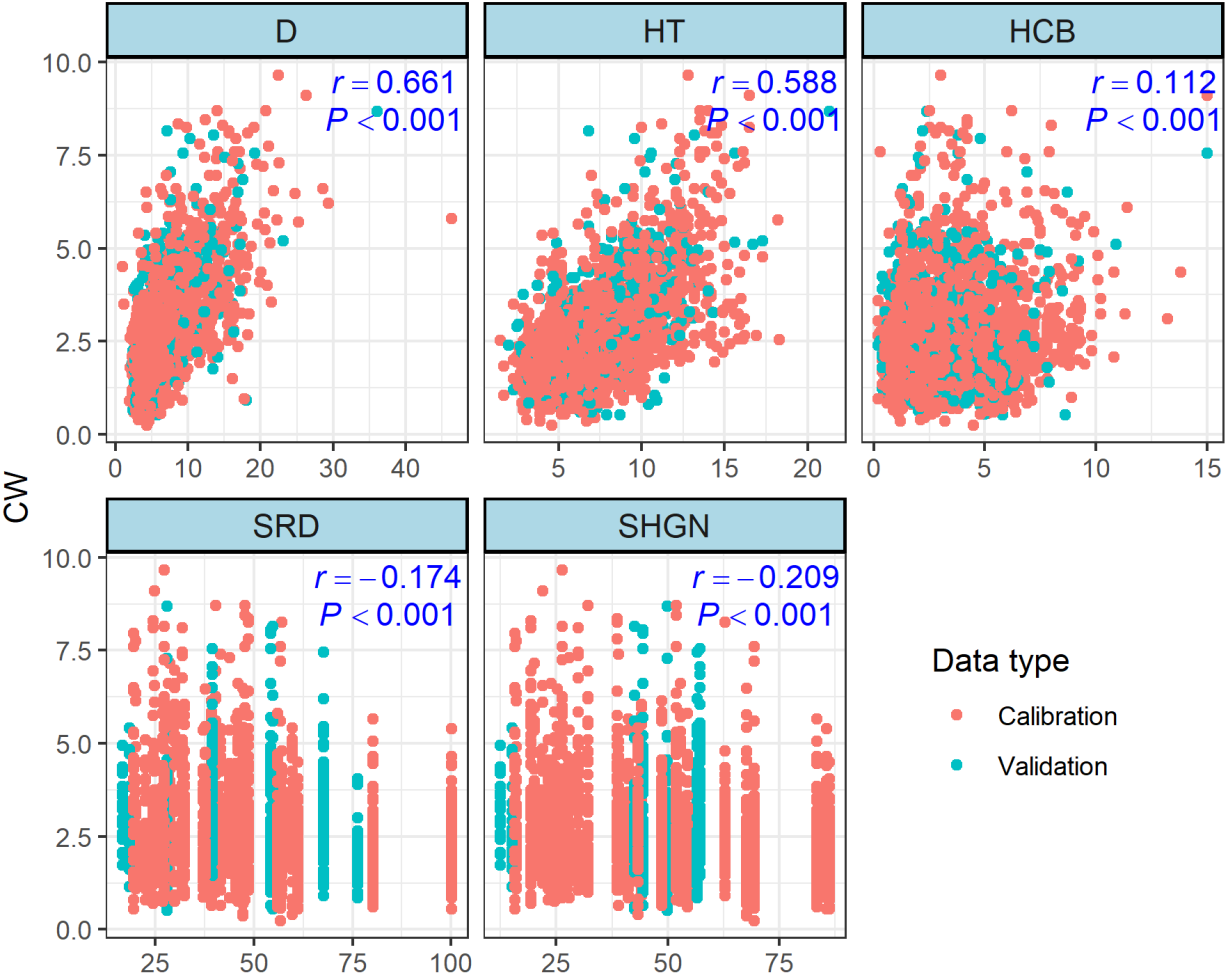
The relationships among crown width (CW) with diameter at breast height (D), height to crown base (HCB), tree height (TH), sum of relative DBH (SRD) and sum of the Hegyi index for fixed number competitors (SHGN).

**Figure 3:**
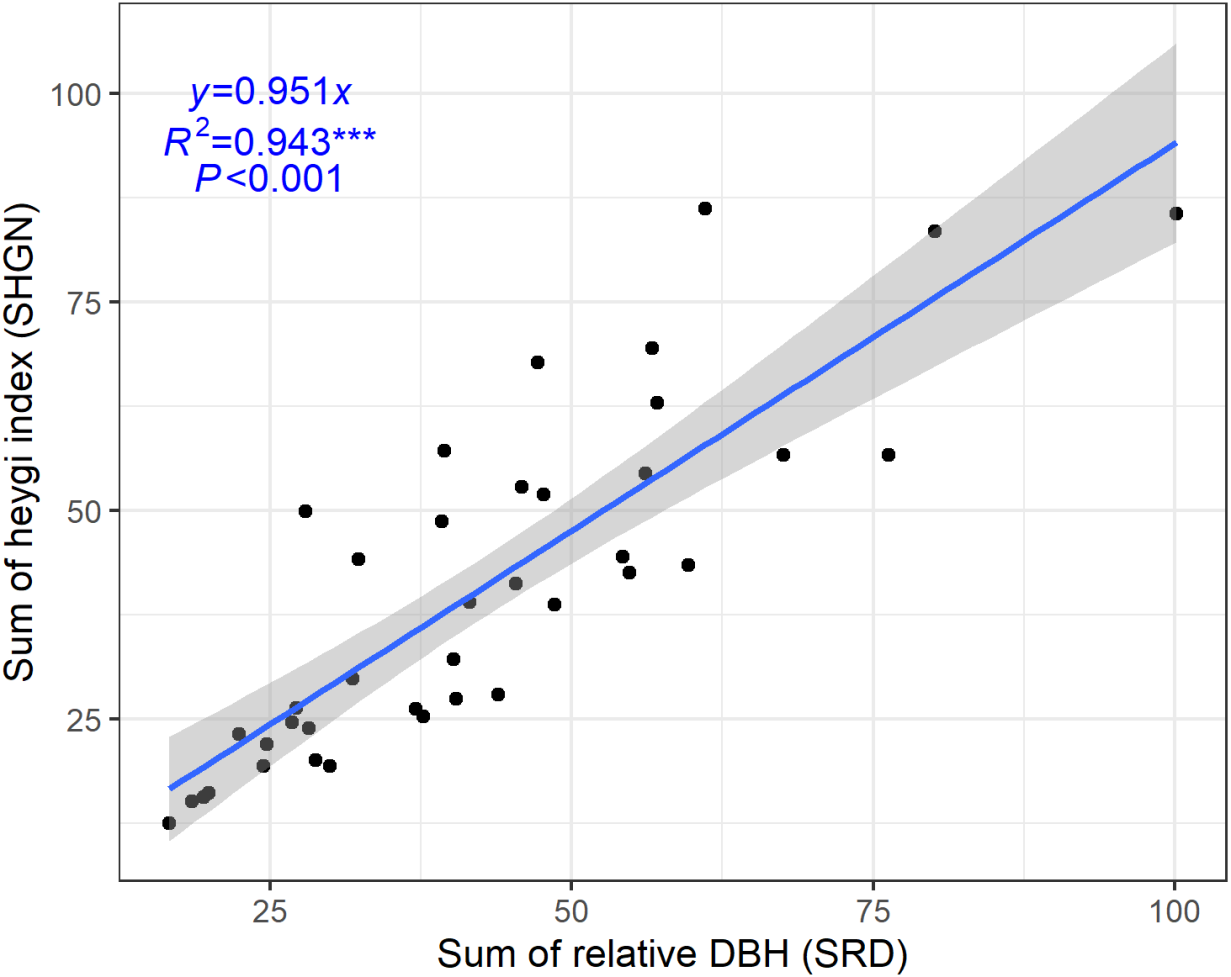
Relationships between sum of relative DBH and sum of Hegyi index.

To avoid the effects of over-parameterization and collinearity in the estimated models, we selected variables with graphical exploration of the data and examination of the correlation statistics, and only those variables displaying a significant contribution to crown width variation were retained (Fig. 2). The selected variables are: tree height (TH), height of crown base (HCB), and sum of relative dbh (SRD) and sum of hegyi index (SHGN) were identified as the optimum distance-independent competition indices and distance-independent competition indices, respectively. Correlation analysis showed that these variables were significantly correlated with the CW (Fig. 3). Therefore, the function was expanded as follows (F2):

(4) \begin{eqnarray*}{\mathrm{CW}}_{ij}=(\phi 1+\phi 3SR{D}_{i}/SHG{N}_{i}+\phi 4T{H}_{ij}+\phi 5HC{B}_{ij}){D}_{ij}^{\phi 2}+{}_{ij}\end{eqnarray*}

where TH_*ij*_, HCB_*ij*_represent the tree height (m), height of crown base (cm) of the *j*th tree in the *i*th plot, SRD_*i*_/SHGN_*i*_ represents the distance-independent/distance-dependent competition index of the *i* th plot; *ϕ*1, *ϕ*2, *ϕ*3, *ϕ*4, and *ϕ*5 are formal parameters. The spatially non-explicit and spatially explicit generalized CW models were obtained and are displayed in Eqs. (5) and (6), respectively. The goodness-of-fit statistics of the generalized CW models are shown in Table 6.

(5) \begin{eqnarray*}{\mathrm{CW}}_{ij}& =(0.901-0.001{\mathrm{SRD}}_{i}+0.066{\mathrm{TH}}_{ij}-0.042{\mathrm{HCB}}_{ij}){\mathrm{D}}_{ij}^{0.441}+{}_{ij}\end{eqnarray*}

(6) \begin{eqnarray*}{\mathrm{CW}}_{ij}& =(0.995-0.003{\text{SHGN}}_{i}+0.065{\mathrm{TH}}_{ij}-0.038{\mathrm{HCB}}_{ij}){\mathrm{D}}_{ij}^{0.434}+{}_{ij}\end{eqnarray*}

**Figure 4:**
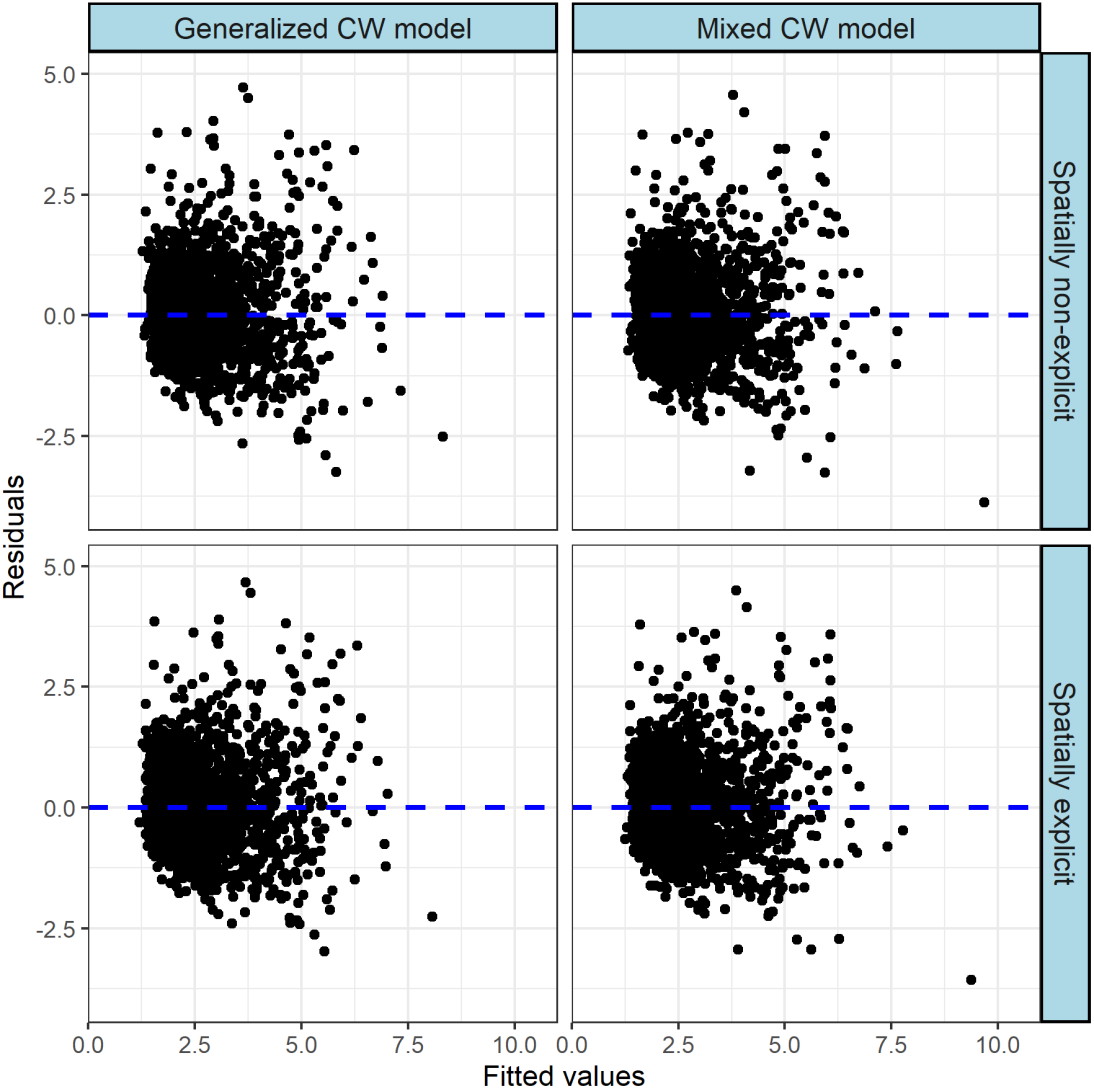
Standardized residuals plots of the models.

The multiple model performance criteria confirmed that the both generalized CW models Eqs. (5) and (6) show a substantial improvement compared with basic CW model Eq. (3) when the covariates are added. Meanwhile, the spatially explicit generalized CW-D model Eq. (6) performed better than spatially non-explicit model Eq. (5) (Table 7). Figure 4 also demonstrates that heteroscedasticity in the residuals was reduced by generalized models, and heteroscedasticity in the residuals for the spatially explicit generalized model was smaller than that for the spatially non-explicit generalized model.

**Table 7:** Parameter estimate, variance components, and fit statistics of CW models in the analysis.

Item	Parameters	Generalized CW model		Mixed CW model	
		Spatially non-explicit Eq. (5)	Spatially explicit Eq. (6)	Spatially non-explicit (8)	Spatially explicit Eq. (9)
Fixed parameters	*ϕ*1	0.901^***^	0.995^***^	0.967^***^	1.057^***^
	*ϕ*2	0.441^***^	0.434^***^	0.417^***^	0.410^***^
	*ϕ*3	−0.001^**^	−0.003^***^	−0.001^**^	−0.003^***^
	*ϕ*4	0.066^***^	0.065^***^	0.059^***^	0.057^***^
	*ϕ*5	−0.042^***^	−0.038^***^	−0.028^***^	−0.022^***^
Variance components	*σ* ^2^	0.763	0.743	0.733	0.713
	${\sigma }_{u2}^{2}$	−	−	0.002	0.002
	${\sigma }_{u5}^{2}$	−	−	0.0004	0.0005
	*σ*_*u*2*u*5_	−	−	−0.0009	−0.001
Calibration	AIC	5709	5651	5645	5584
	${R}_{a}^{2}$	0.520	0.533	0.539	0.552
	RMSE	0.872	0.861	0.855	0.843
	MAE	0.658	0.651	0.644	0.638
	MAPE	0.761	0.742	0.731	0.711
Validation	${R}_{a}^{2}$	0.441	0.450	0.455	0.461
	RMSE	1.018	1.010	1.011	1.006
	MAE	0.750	0.745	0.742	0.739
	MAPE	1.036	1.020	1.023	1.011

**Figure 5:**
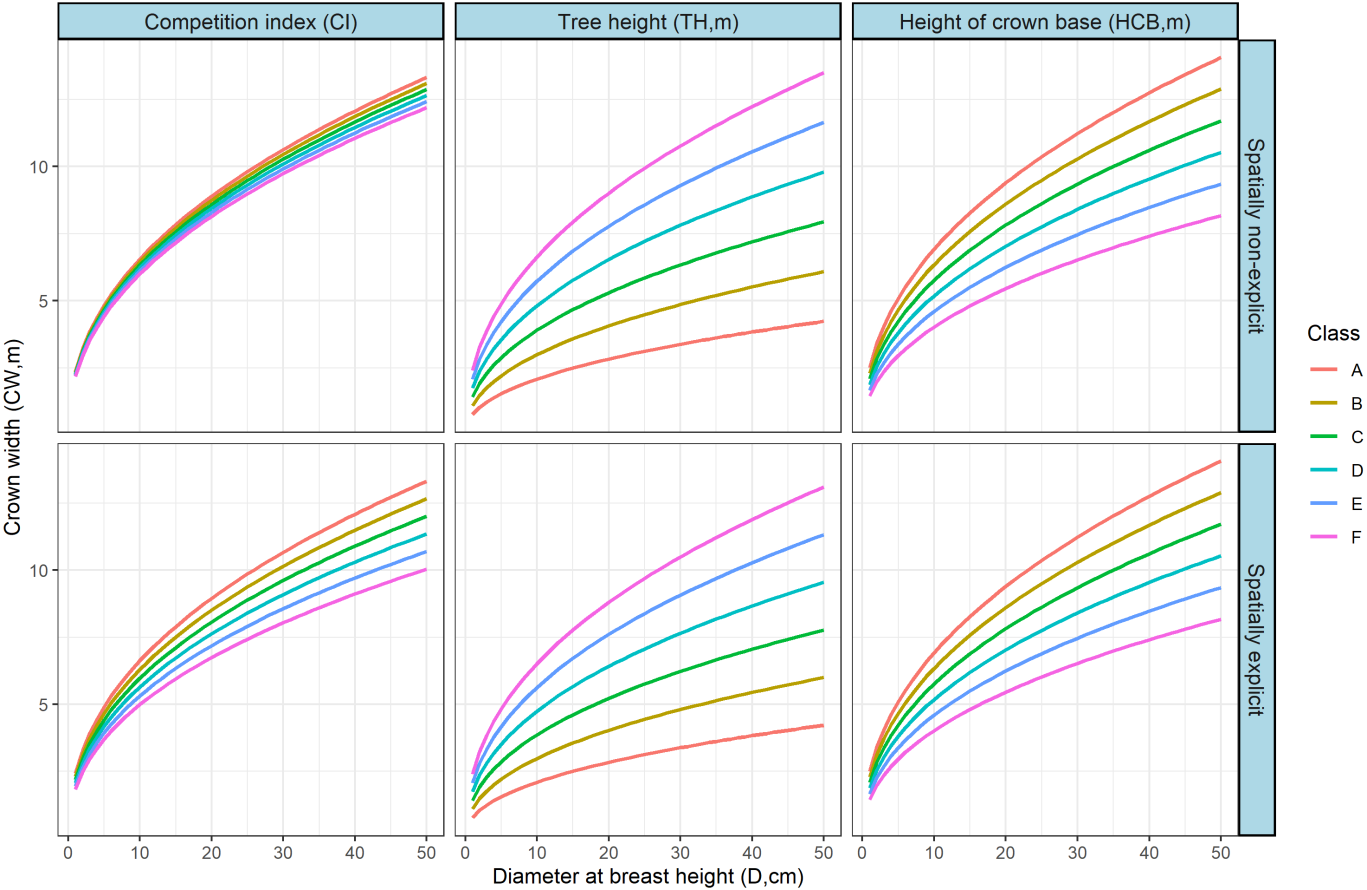
Effects of the corresponding variables on the on the CW-D relationship were simulated by CW-D curves using the generalized CW-D model in Table 6 (Eqs. (5) and (6)). CI index roughly divided into six equal intervals, A: 10, B: 50, C: 90, D:130, E:170, F:210; Tree height, A: 1, B: 6, C: 11, D:16, E:21, F:26; height of crown base, A: 0, B: 5, C: 10, D:15, E:20, F:25. The TH = 25, CI = 45, HCB = 4 were used for other variables rather than the variable shown varying (lowest to highest range in the observed data).

The effects of HCB, TH and CI on the CW-D relationship were simulated by CW-D curves (Fig. 5) using the generalized CW-D model (Eqs. (5) and (6)). The variables of interest were roughly divided into six equal intervals, and the simulation demonstrated that the HCB, TH, and CI had more considerable contribution to the CW-D models, the differences of CW were greater with the increase of DBH, and each of HCB, TH, and CI significantly affected the initial CW-D relationship.

### Mixed-effects CW model

There are 31 potential different combinations of random effects for Eq. (4) considering all independent variables and the intercept in the generalized model when species is included as random effect. Fitted to the data, 25 of the mixed-effects model alternatives reached convergence, with Eq. (7) yielding the smallest AIC (AIC = 5645) for the spatially explicit mixed-effects model, and 23 of the mixed-effects model alternatives reached convergence, with Eq. (7) yielding the smallest AIC (AIC = 5584) for the spatially non-explicit mixed-effects model.

(7) \begin{eqnarray*}{\mathrm{CW}}_{ij}=[\phi 1-\phi 3SR{D}_{i}/SHG{N}_{i}+\phi 4T{H}_{ij}+(\phi 5+u{5}_{i})HC{B}_{ij}]{D}_{ij}^{(\phi 2+u{2}_{i})}+{}_{ij}\end{eqnarray*}

where *ϕ*1 − *ϕ*5 are fixed-effects parameters, u2_*i*_, u5_*i*_ are random-effects parameters generated by tree species. After the determination of parameter effects and error variance–covariance structure, the final mixed-effects models were proposed in Eqs. (8) and (9). The residual plots and goodness-of-fit statistics are shown in Fig. 4 and Table 5, respectively.

(8) \begin{eqnarray*}{\mathrm{CW}}_{ij}=[0.967-0.001SR{D}_{i}+0.059T{H}_{ij}+(-0.028+u{5}_{i})HC{B}_{ij}]{D}_{ij}^{0.417+u{2}_{i}}+{}_{ij}\end{eqnarray*}

where

\begin{eqnarray*}{\mathrm{u}}_{i}= \left[ \begin{array}{@{}c@{}} \displaystyle u{2}_{i}\\ \displaystyle u{5}_{i} \end{array} \right] \sim N \left\{ \left[ \begin{array}{@{}c@{}} \displaystyle 0\\ \displaystyle 0 \end{array} \right] ,\psi = \left( \begin{array}{@{}cc@{}} \displaystyle 0.002&\displaystyle -0.0009\\ \displaystyle -0.0009&\displaystyle 0.0004 \end{array} \right) \right\} ,{}_{ij}\sim N \left( 0,{R}_{ij}=0.733{G}_{ij}^{0.5}{I}_{ni}{G}_{ij}^{0.5} \right) ,\nonumber\\\displaystyle var \left( {}_{ij} \right) =0.733{D}_{ij}^{-3.012} \end{eqnarray*}

and

(9) \begin{eqnarray*}{\mathrm{CW}}_{ij}=[1.057-0.003SHG{N}_{i}+0.057T{H}_{ij}+(-0.022+u{5}_{i})HC{B}_{ij}]{D}_{ij}^{(0.410+\mathrm{u}{2}_{i})}+{}_{ij}\end{eqnarray*}

where

\begin{eqnarray*}{\mathrm{u}}_{i}= \left[ \begin{array}{@{}c@{}} \displaystyle u{2}_{i}\\ \displaystyle u{5}_{i} \end{array} \right] \sim N \left\{ \left[ \begin{array}{@{}c@{}} \displaystyle 0\\ \displaystyle 0 \end{array} \right] ,\psi = \left( \begin{array}{@{}cc@{}} \displaystyle 0.002&\displaystyle -0.001\\ \displaystyle -0.001&\displaystyle 0.0005 \end{array} \right) \right\} ,{}_{ij}\sim N \left( 0,{R}_{ij}=0.713{G}_{ij}^{0.5}{I}_{ni}{G}_{ij}^{0.5} \right) ,\nonumber\\\displaystyle var \left( {}_{ij} \right) =0.713{D}_{ij}^{-2.722}. \end{eqnarray*}

We found that the both mixed-effects CW models Eqs. (8) and (9) show a substantial improvement compared with corresponding generalized CW model Eqs. (5) and (6) when species is included as random effect. We found that the addition of random parameters in could significantly improve the predictive ability for crown width. Meanwhile, the spatially explicit mixed CW-D model Eq. (9) performed better than the spatially non-explicit model Eq. (8) (Table 5).

### Relationship between SHGN and SRD

Considering the importance of competition for crown width development and modelling, SHGN and SRD were selected for the analyses of linear regression to quantify competition. SHGN had a significant positive linear correlation with SRD (Fig. 3, *R*^2^ = 0.943 and *P* < 0.001).

## Discussion

CW-D models are commonly used to predict the crown width of trees (Buchacher & Ledermann, 2020). The models proposed in the current study explain the greatest portion of crown width variation and fully confirm that crown width and DBH are strongly correlated. In our study, the Power model proved more effective in delineating the basic CW-D relationship for studied secondary forests than other candidate models. The Power model has been widely used to describe tree allometric relationships, especially for modelling CW-D relationships (Russell & Weiskittel, 2011; Sharma et al., 2017; Raptis et al., 2018). Herein, the Power model was the most flexible model, as it is easily linearized and expanded to a generalized model (Raptis et al., 2018). In addition, the Power function is a two-parameter function, which is easier to fit and more quickly achieves convergence during data processing than other candidate three-parameter functions. Therefore, the Power function is recommended as the basic model to be applied for accurate predictions of the CW-D relationship for dominant tree populations.

The incorporation of DBH as the only predictive variable assumes that trees of the same stem diameter also have the same mean crown dimensions. However, the development of crown width is influenced by numerous factors. In many instances, such CW-D relationships are complex nonlinear processes that are difficult to describe using ordinary parameter models, which may overestimate crown width for crowded stands and underestimate crown width for sparse stands (Sharma et al., 2017; Buchacher & Ledermann, 2020). Notably, this is the case regardless of competition inside the stand (Raptis et al., 2018). Several studies have reported that tree crown width decreases with increasing competition. This is biologically plausible and interpretable, as competition or spacing determines crown shape and size, and crowding in a stand results in trees growing taller with smaller crowns and narrower crown widths (Sharma, Vacek & Vacek, 2016), and introduction of competition indices could significantly improve the performance of CW models.

As indicated by studies modelling the crowns of various species (*e.g.*, Fu et al., 2013; Sharma, Vacek & Vacek, 2016), the tree size parameters HCB and TH highly correlate with individual tree growth and stand dynamics, and emerge as important covariate predictors in our CW-D model. This is because HCB is closely related to crown dimensions and significantly affects tree vigor (Sharma, Vacek & Vacek, 2016; Fu et al., 2017), while TH can represent the relative dominance among the trees. Crown recession is significantly affected by light availability at the base of tree crowns (Sorrensen-Cothern, Ford & Sprugel, 1993), and occurs when branches at the crown base die back, resulting in a larger HCB and narrower crown. At the same time, a larger TH would be indicative of the tendency to seek more light resources for the crown, especially the base of the crown, to prevent crown recession. Because of these characteristics, HCB and TH have frequently been used as covariate predictors in various forest models, including crown width models (Sharma et al., 2017). In the current study, the tree and stand variables DBH, TH, HCB and CI, as covariate predictors, contributed significantly to the higher prediction accuracy of the crown width model (Figs. 4 and 5).

In addition, both distance-dependent and distance-independent CIs were evaluated for their effects on crown width modelling and predictions. Several studies have also documented that distance-dependent CIs were superior to distance-independent ones, especially in mixed-species stands (Contreras, Affleck & Chung, 2011; Quinonez-Barraza et al., 2018; Pommerening, 2008; Sharma, Vacek & Vacek, 2016). Our results support their conclusion in that both the spatially explicit and non-spatially explicit generalized or mixed-effects CW models greatly reduce bias and improve precision. However, the spatially-explicit CW models performed better than corresponding non-spatially explicit models, which indicates that spatially explicit models can be more appropriate for the description of individual tree growth dynamics than spatially non-explicit ones (Fig. 4 and Table 7), as has been found in several similar studies (Sharma, Vacek & Vacek, 2016; Yang & Huang, 2017). This study further explored the correlation between distance-dependence (Hegyi index) and distance-independence (Sum of the relative DBH), and established a highly significant correlation between them. Ease of use in forestry practice should be an important concern of the analysts towards the development of such CW models (Raptis et al., 2018). Therefore, we suggest that our spatially non-explicit generalized CW-D model can be applied for precise predictions of crown width, as it does not require spatially explicit competition measures, which can be computationally complex and difficult (Sharma, Vacek & Vacek, 2016).

Mixed-effects models are widely applied in forestry modeling and are useful tools for accommodating hierarchical structured data, repeated measures data, and spatially correlated data, and always provide a more stable estimate (Zhang et al., 2019). More importantly, their inherent flexibility allows for the development of a unique variance–covariance structure, which is restricted in traditional nonlinear regression (Fu et al., 2013). In this study, our developed mixed-effects CW models for secondary forests show strong practicability and operability in predicting CW and had significantly improvement in model performance, either than generalized CW or basic models without random effects. Therefore, the models we developed can provide a simple and effective approach for predicting CW and we strongly recommend their future application.

## Conclusions

In this study, a simple power function was chosen to expand a basic CW model through the integration of tree size variables, stand variables, and competition measures. Diameter at breast height, height to crown base, tree height, and competition indices made the largest contributions to the models, and competition was a key factor in CW prediction. The sum of relative DBH was identified as the optimal distance-independent competition index (CI) and as a covariate predictor for spatially explicit CW models, and the sum of the Hegyi index for fixed number competitors was the optimal distance-dependent CI for spatially non-explicit CW models. Both spatially non-explicit and spatially explicit mixed-effects CW models were subsequently developed for secondary forests in the studied region. Spatially explicit models exhibited a significantly greater effect on CW than spatially non-explicit ones. However, spatially explicit models can be replaced by corresponding spatially non-explicit models due to the small differences in the fit statistics, and because the two competition indices are significantly linearly correlated. In summary, the newly developed spatially non-explicit and spatially explicit mixed-effects CW models are not only valuable for predicting tree CW, but also provide an essential tool for forestry inventory and management of secondary forests in the studied region.

## Supplemental Information

10.7717/peerj.13105/supp-1 Supplemental Information 1 Raw data

## Additional Information and Declarations

### Data Availability

The following information was supplied regarding data availability: Data are available at GitHub: https://github.com/Zongzheng/resdata/blob/main/karstCW.csv.
